# ChemMaps.com v2.0: exploring the environmental chemical universe

**DOI:** 10.1093/nar/gkad380

**Published:** 2023-05-17

**Authors:** Alexandre Borrel, Mike Conway, Sue Z Nolte, Aswani Unnikrishnan, Charles P Schmitt, Nicole C Kleinstreuer

**Affiliations:** Inotiv, RTP, NC, 27560, USA; NIH/NIEHS/ODS, RTP, NC 27709, USA; NIH/NIEHS/ODS, RTP, NC 27709, USA; Inotiv, RTP, NC, 27560, USA; NIH/NIEHS/ODS, RTP, NC 27709, USA; NIH/NIEHS/DTT/NICEATM, RTP, NC 27709, USA

## Abstract

Access to computationally based visualization tools to navigate chemical space has become more important due to the increasing size and diversity of publicly accessible databases, associated compendiums of high-throughput screening (HTS) results, and other descriptor and effects data. However, application of these techniques requires advanced programming skills that are beyond the capabilities of many stakeholders. Here we report the development of the second version of the ChemMaps.com webserver (https://sandbox.ntp.niehs.nih.gov/chemmaps/) focused on environmental chemical space. The chemical space of ChemMaps.com v2.0, released in 2022, now includes approximately one million environmental chemicals from the EPA Distributed Structure-Searchable Toxicity (DSSTox) inventory. ChemMaps.com v2.0 incorporates mapping of HTS assay data from the U.S. federal Tox21 research collaboration program, which includes results from around 2000 assays tested on up to 10 000 chemicals. As a case example, we showcased chemical space navigation for Perfluorooctanoic Acid (PFOA), part of the Per- and polyfluoroalkyl substances (PFAS) chemical family, which are of significant concern for their potential effects on human health and the environment.

## Graphical Abstract

**Graphical Abstract:**
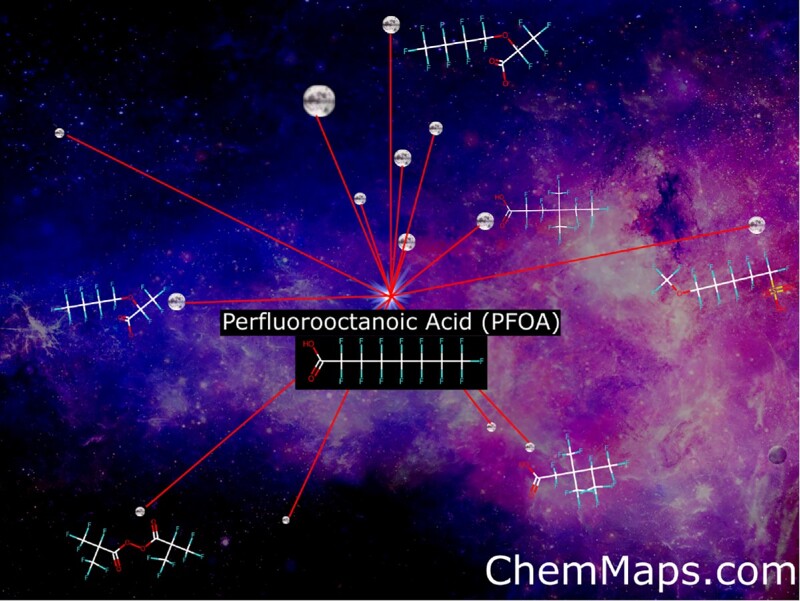
Representation of the perfluorooctanic acid (PFOA) and its neighborhood chemicals on the space computed using over 1 million chemicals from the Distributed Structure-Searchable Toxicity (DSSToxMap).

## INTRODUCTION

Easily navigating chemical space is a current challenge today due to the rapid growth of chemical databases and the multiple steps required to represent multidimensional data in a navigable space. Chemography, defined as the field for navigating chemical space (1,2), is facing scientific cyberinfrastructure challenges to improve navigation tools as well as a need to leverage cheminformatics approaches to define the space that typically rely upon complex projection techniques (3). Typically, a chemical space is defined based on a large set of chemicals projected into two or three dimensions, where relative distances between chemicals are a function of their similarity.

Navigating in chemical space has several established applications in drug discovery. It can be used to identify analogs (4), guide drug optimization (5) or more broadly to expand the drug space by observing and interpreting chemical similarity in a large chemical universe (6–8). However, little attention has been given to navigating the environmental chemical space, i.e. chemical space defined using chemicals found in the environment such as industrial chemicals, pesticides, food additives, personal care product ingredients, and contaminants of emerging concern. Navigating within this space could have major applications in regulatory decision frameworks to identify structural analogues for data-poor chemicals that could support risk assessments, research projects involving non-targeted analysis to define chemicals that may be present in the exposome (9), or read-across where similar chemical properties are used to fill data gaps for chemicals of interest (10), to name a few.

In 2018, we developed ChemMaps.com, a web-based tool inspired by Google Maps, to navigate the chemical space (11). We focused the first version of the tool on the drug space, by projecting the DrugBank database (12) that included approved drugs and drugs-in-development. Subsequently, we developed an initial environmental chemical space using the U.S. EPA Toxic Substances Control Act (TSCA) inventory (https://www.epa.gov/tsca-inventory) of >40 000 chemicals. Here we present the development of ChemMaps.com v2.0., which substantially extends the scope of the first version to incorporate the U.S. Environmental Protection Agency's Distributed Structure-Searchable Toxicity (DSSTox) database (13); with over 1 million chemicals, this is the world's largest publicly available curated structural database for environmental chemicals. In ChemMaps.com v2.0, we have also mapped the rich *in vitro* assay data sets from the Tox21 and ToxCast high-throughput screening programs, covering thousands of cellular and molecular targets relevant to toxicological modes of action (14). In addition to the chemical space and annotated data expansion, ChemMaps.com v2.0 provides new functionalities based on user-identified needs.

## MATERIALS AND METHODS

### Expansion of chemical space

Originally ChemMaps.com was developed to navigate in two maps, called the DrugMap and the EnvMap, computed respectively from (i) drugs and drugs-in-development available in the DrugBank database (12) and (ii) chemicals included in the TSCA inventory. ChemMaps.com v2.0 now includes a vastly extended environmental chemicals universe divided into three maps. The largest map was developed by incorporating the U.S. Environmental Protection Agency's Distributed Structure-Searchable Toxicity (DSSTox) database, the world's largest publicly available curated database for environmental chemicals with over 1 million structures (15), and is called DSSToxMap. The DSSToxMap included all the chemicals included in all of the other maps available in ChemMaps.com. We developed two additional sub-maps: one with all 14629 identified per- and polyfluorinated substances (PFAS) structures downloaded on the EPA chemical dashboard (13,16) called PFASMap, and one with the 8236 chemicals that were tested in the Tox21 and ToxCast high-throughput screening programs called Tox21Map (17). These two subgroups of chemicals were chosen due to their prevalence in the environment and increasing concern over ecological and human health impacts, and their wealth of data on mechanistically informative targets, respectively. We updated the DrugMap with the latest version of the DrugBank (v5.1.10, release 2023-01-04).

Maps were computed using an updated version of the same approach originally developed in ChemMaps v1.0 (11), i.e. using a set of non-correlated and informative (descriptor variance not null) 1D, 2D and 3D molecular descriptors computed using RDKIT (version 2021). Chemicals are projected into the space using a combination of two principal component analyses calculated from the 1D and 2D descriptors for the first two dimensions and 3D descriptors for the third dimension of the space. We developed a python library called CompDesc (v1.0.3), made available to compute molecular descriptors using RDKIT (https://test.pypi.org/project/CompDesc/).

### Chemical feature projections

Chemicals are represented in the space by a star or a planet, depending on the level of information available. For the DrugMap, stars are used to represent approved and withdrawn drugs and planets are used to represent drugs-in-development. For the environmental chemical maps (DSSToxMap, PFASMap and Tox21Map), stars are used if chemicals have oral acute toxicity class according to the United Nations Globally Harmonized System of Classification and Labelling of Chemicals (UN GHS) available (18,19). In addition to the shape representation, up to five features can be chosen by users and used to color chemicals on the maps. DrugMaps features include experimental physicochemical properties available in the DrugBank database, and for DSSToxMap, PFASMap and Tox21Map predicted physicochemical properties computed using OPERA v2.8 (20).

### Chemical bioactivities

We have mapped the rich *in vitro* assay data sets from the Tox21 and ToxCast high-throughput screening programs, covering thousands of cellular and molecular targets, to the Tox21Map in 3D chemical space. Users can select specific assays or groups of assays based on their target to see on the Tox21Map chemical activities and the most active assays by chemicals. We also provide to users an interactive spreadsheet to navigate data and the option to project on each chemical its most potent activity, as represented by the lowest AC50 and corresponding assay target. We used the curated version of the HTS data processed using the US EPA’s tcpl R package (21) and the National Toxicology Program Interagency Center for the Evaluation of Alternative Toxicological Methods (NICEATM) Integrated Chemical Environment curation and annotation workflow (22–24).

### User-defined chemicals

In ChemMaps.com v2.0, users can upload up to 100 of their own chemicals to project onto a chosen map. Input options include SMILES format, CASRN ID or DTXSID (unique structural identifier from US EPA’s DSSTox database). Chemicals will be prepared and projected on the fly in three steps to allow users to control and refine their input. To save computational time, each chemical uploaded is saved internally in our database. On the map, chemicals are represented using a rocket and are assigned an ID that can used in the search bar.

### Webserver navigation

Since all information and coordinates of the molecules are pre-computed, browsing does not require computational skills. The DSSToxMap that includes more than 1 million chemicals is loaded by subset, each comprising around 10 000 chemicals centered on the chemicals of interests, i.e. preselected chemicals or those uploaded by users. ChemMaps.com v2.0 was developed to work on commonly used web-browsers and tested for Firefox 111.0.1, Chrome 105.0.5195.102, and Edge 104.0.1293.70 and requires the WebGL JavaScript API as a dependence.

### Webserver development

The webserver was developed using Django in Python 3.9 on a Linux server. Data is stored in a PostgreSQL database used to store molecular descriptors, coordinates, and the 20 closest neighbors for each chemical and corresponding prepared structure. More than one million chemical entries are included in the database.

## RESULTS

### Application for PFOA

Here we demonstrate the use of ChemMaps.com v2.0 to explore the chemical space around perfluorooctanoic acid (PFOA). The chemical PFOA belongs to the per-and polyfluoroalkyl substances (PFAS) chemicals family, also called ‘forever chemicals’. These chemicals contain a least one polyfluoroalkyl chain that gives them particular resistance properties and are used in consumer products and industry (25). PFAS, and particularly PFOA, are high-concern chemicals since they are found in the blood of >97% of Americans, and there is strong emerging evidence that they can contribute to a variety of adverse health effects, including altered immune and thyroid function, liver disease and cancer (26).

**Figure 1.**
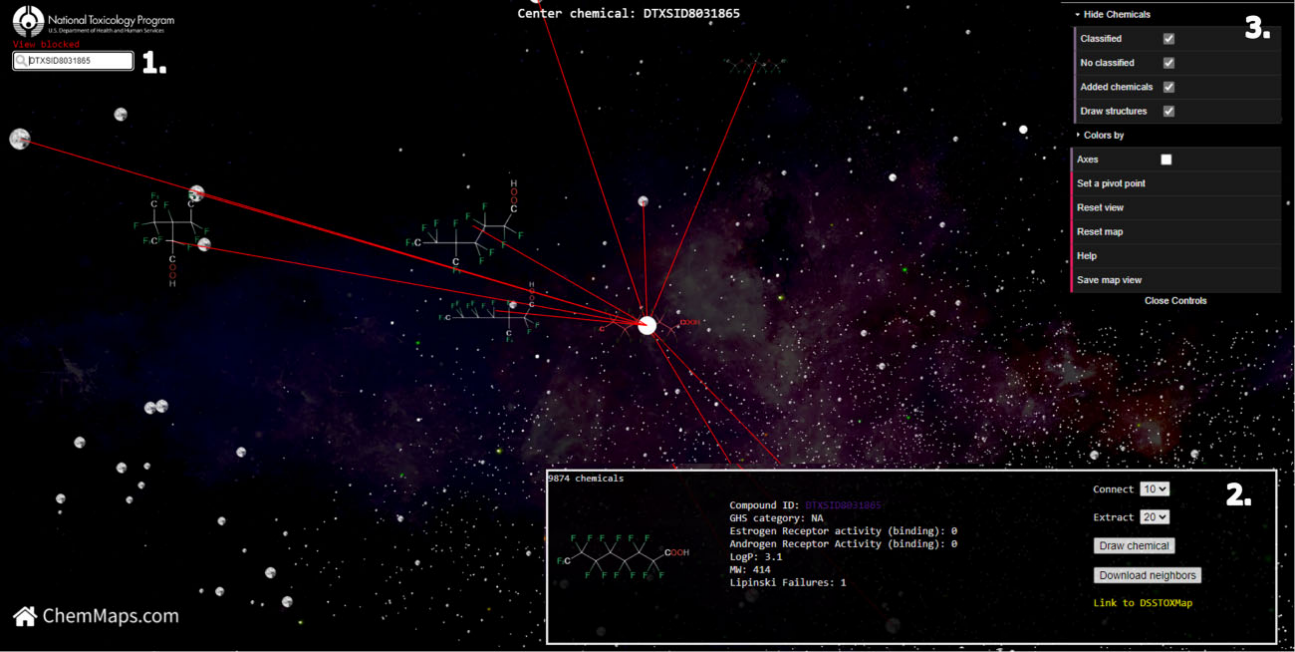
Screenshot of the DSSToxMap centered on PFOA (DTX8031865). The navigation windows include a search bar (**1**) to search and centre the view based on a chemical; an interactive info panel (**2**) where chemicals information are updated in real time during the navigation and also links to the EPA chemicals dashboard, and a navigation panel (**3**) that allows users to select a subset of chemicals and change chemicals colour based on a set of previous selected features.

**Figure 2.**
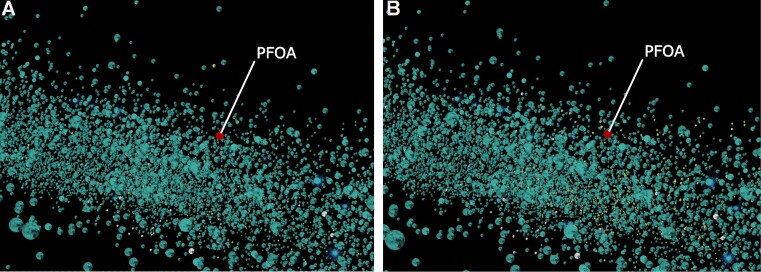
Screenshot of PFOA on the PFASMap colored based on predictive ER agonist (**A**) and AR antagonist activity (**B**). Chemicals predicted ER agonist or AR antagonist are represented in yellow.

**Figure 3.**
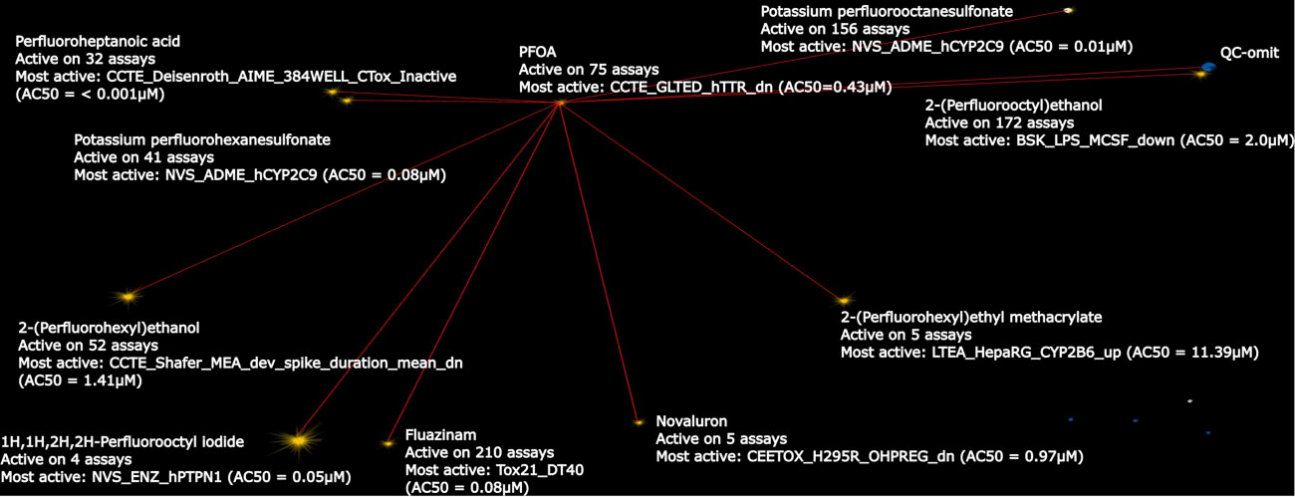
Screenshot of the position of PFOA on the Tox21Map. For each chemical the most active assay is represented with the number of positive assays in the Tox21/ToxCast program. The ten first neighbors are represented.

First, we searched PFOA (DTXSID8031865) on the DSSToxMap, Figure 1. PFOA does not have a measured oral acute toxicity value but its ammonium form, ammonium perfluorooctanoate, has been studied and is classified as GHS 4 (harmful if inhaled). The chemical space around PFOA is an area where the density and diversity of chemicals is low. Most of the direct neighbors of PFOA are also PFAS chemicals. A variety of analysis features may be projected onto the space. For example, PFOA and its closest neighbors (up to 20) fail the Lipinski rule by one property, which makes them more likely to be absorbed by the body and be bioaccumulative. We then explored PFOA on the PFASMap including only PFAS chemicals, Figure 2. Most of its neighbors’ chemicals on the DSSToxMap are the same as its neighbors on the PFASMap, and we can examine whether neighboring chemicals are predicted to be androgen receptor antagonist or estrogen receptor agonist which can help to identify endocrine disruption effects of these chemicals, (27,28), Figure 2. The neighborhood of PFOA includes only few chemicals with an acute tox GHS classification (most in class 4 to 5), demonstrating that a broad range of these ‘forever chemicals’ remain untested in traditional toxicity studies. Finally, we explored the neighborhood of PFOA on the Tox21Map, where the lowest AC50 and the count of active assays from the Tox21 high throughput screening program for each chemical are mapped, Figure 3. PFOA is reported active in 75 assays from the Tox21/ToxCast program and is the most active in an assay that targets transthyretin (TTR) (CCTE_GLTED_hTTR_dn) with an AC50 equal to 0.43 μM. TTR is one of the major transport proteins responsible for binding to and transporting thyroid hormones to the necessary tissues. A detailed view of available toxicological information and specific assay results by chemical can be explored on the US EPA CompTox Chemicals dashboard (13) which is linked from ChemMaps v2.0 via the chemical ID in the chemical information panel. By exploring the neighborhood of PFOA we noticed that surrounding chemicals are most active in assays that impact crucial pathways such as steroid hormone metabolism (NVS_ADME_hCYP2C9), xenobiotic metabolism (LTEA_HepaRG_CYP2B6_up), hepatic metabolism (CCTE_Deisenroth_AIME_384WELL_CTox_Inactive_dn) and functional neural network activity (CCTE_Shafer_MEA_dev_spike_duration_mean_dn) looking specifically at the five closest chemicals. Exploring this space using this visual tool gives potential activity clues for assays and endpoints that have not yet been tested, and can assist read-across analyses where similar chemicals profiles can fill testing gaps.

## DISCUSSION

The expansion of the ChemMaps.com v2.0 space to an unprecedented number of environmental chemicals, and the ability to project user-defined chemicals, has tremendous utility to quickly visualize and explore chemicals of potential concern for impacts on human health and the environment, and to guide future research directions. The ChemMaps.com v2.0 website is free and open to all users, there is no login requirement, and it is available at https://sandbox.ntp.niehs.nih.gov/chemmaps/.

## DATA AVAILABILITY

DSSTox chemicals are freely available on the EPA chemical dashboard at https://comptox.epa.gov/dashboard. The code developed to build ChemMaps.com v2.0 database is available at https://github.com/ABorrel/chemmaps_data-process. The webserver code is available at https://github.com/ABorrel/chemmaps-InterPred-Bodymap and in FigShare at https://doi.org/10.6084/m9.figshare.22344832.v1.

The ChemMaps.com v2.0 website (https://sandbox.ntp.niehs.nih.gov/chemmaps/) is free and open to all users and there is no login requirement.
